# Supplementary material for: Long-Term Protein Synthesis with PURE in a Mesoscale Dialysis System
Source: ACS Synth Biol. 2025 Jan 6;14(1):290–5. doi: 10.1021/acssynbio.4c00618 (PMC11744927; doi:10.1021/acssynbio.4c00618)
Supplement: Supplementary file 1 — sb4c00618_si_001.pdf [file sb4c00618_si_001.pdf]

Long-term protein synthesis with PURE in a mesoscale dialysis system

Laura Roset Julià †[1,2], Laura Grasemann †[3], Francesco Stellacci[1,2,4], and Sebastian J. Maerkl*[3]

[1]Institute of Materials, School of Engineering, École Polytechnique Fédérale de Lausanne, Lausanne 1015, Switzerland
[2]Swiss National Center for Competence in Research (NCCR) Bio-Inspired Materials, University of Fribourg, Fribourg 1700, Switzerland
[3]Institute of Bioengineering, School of Engineering, École Polytechnique Fédérale de Lausanne, Lausanne 1015, Switzerland
[4]Global Health Institute, École Polytechnique Fédérale de Lausanne, Lausanne 1015, Switzerland

Supplementary Information

†These authors contributed equally to this work
*Correspondence: sebastian.maerkl@epfl.ch

A

GFP concentration [mg/mL]	Fluorescence [RFU] 1	Fluorescence [RFU] 2	Average RFU
0.005	522	505	514 ± 12
0.01	1541	1514	1527 ± 19
0.05	18064	18362	18213 ± 210
0.1	40624	41009	40816 ± 272
0.5	OVERFLOW	OVERFLOW	OVERFLOW

B

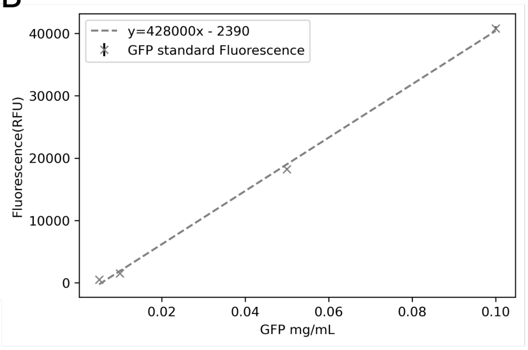

Figure S1: (A) Relative Fluorescence Units (RFU) of the calibration standards, measured in duplicate. The first two columns contain the measured data, while the third column is an average of the two, used to perform the linear regression. (B) Plot of the linear regression (grey dashed line) and the averaged RFU of the calibration standards (marked with a cross), with the measured standard deviation.

Sample	Fluorescence [RFU] 1	Fluorescence [RFU] 2	Final Concentration [mg/mL]
REP1 – PURE dialysis refill	41119	43641	1.05 ± 0.04
REP2 – PURE dialysis refill	48803	45474	1.16 ± 0.05
REP1 – PURE dialysis standard	15254	16375	0.43 ± 0.02
REP2 – PURE dialysis standard	11042	11554	0.32 ± 0.01
REP1 – Lysate dialysis refill	8011	8953	0.25 ± 0.02
REP2 – Lysate dialysis refill	12380	13366	0.36 ± 0.02
REP1 – Lysate dialysis standard	7970	8084	0.24 ± 0.00
REP2 – Lysate dialysis standard	10097	10280	0.29 ± 0.00
GFP 0.475 mg/mL control	23309	22756	0.59 ± 0.01

Table S1: Endpoint measure of the relative fluorescence units (RFU) in technical duplicates (first and second columns) of both replicates for each experiment. The last column is the averaged quantification of the duplicates using the calibration curve presented in Figure 1. The standard deviation results from the technical duplicates.

Plasmid Sequence

CCGGTGATGCCGGCCACGATGCGTCCGGCGTAGAGGATCGAGATCGATCTCGATCCCGCGAAATTAATACGACTCACTATAGGGGAATTGTGAGCGGATAACAATTCC

CCTCTAGAAATAATTTTGTTTAACTTTAAGAAGGAGATATACATATGAAACATCATCATCACCACCATCATCACCACCACCATCACCATCATGGCGCCTCTATGGATTA

CAAAGACCATGATGGTGATTACAAGGATCATGATATTGATTATAAAGATGATGATGATAAAGGTTCCGGGTCTGGTGAAAACCTCTACTTTCAAGGGTCGGGATCCAT

GGTTTCCAAAGGAGAAGAACTGTTTACCGGTGTTGTACCAATTCTCGTAGAACTCGATGGAGATGTAAACGGGCATAAATTTTCAGTGCGCGGCGAGGGCGAAGGAGA

TGCCACAAACGGCAAACTGACCCTTAAATTTATTTGCACGACCGGCAAATTACCAGTTCCTTGGCCTACGCTGGTCACCACGCTCACCTATGGGGTATTATGCTTTAGCC

GCTATCCGGATCACATGAAACGCCATGATTTCTTTAAAAGTGCTATGCCAGAAGGTTATGTACAGGAACGCACGATTAGCTTTAAAGATGATGGGACGTATAAAACCC

GCGCCGAGGTAAAATTTGAAGGAGATACCTTAGTAAACCGCATTGAACTCAAGGGGATTGATTTTAAAGAGGACGGTAACATTCTGGGTCATAAACTTGAGTACAACT

TTAACTCACACAACGTTTACATTACCGCGGATAAACAGAAGAACGGTATTAAAGCGTACTTTAAGATTCGCCATAACGTCGAAGATGGCAGTGTTCAGCTGGCCGATC

ATTATCAGCAGAACACGCCGATTGGCGATGGCCCTGTTTTGTTACCGGATAACCATTATTTATCGACTCAGAGCGTCTTAAGTAAAGATCCAAACGAGAAACGCGATC

ACATGGTTCTCTTAGAAGATGTTACCGCCGCCGGCATTACACATGGCATGGATGAACTGTATAAATGATAGGCGGCCGCAGGACTGAATGATATTTTCGAAGCACAAA

AGATTGAGTGGCATGAAGCTAGCGAGAATTTGTATTTTCAAGGTAGTGCTTGGTCGCACCCTCAATTCGAAAAGGGCGGCGGTAGTGGCGGTGGTTCAGGCGGTTCCGC

GTGGAGTCACCCGCAATTCGAGAAAGGCGCTTGATAGCTCGAGCACCACCACCACCACCACTGAGATCCGGCTGCTAACAAAGCCCGAAAGGAAGCTGAGTTGGCTG

CTGCCACCGCTGAGCAATAACTAGCATAACCCCTTGGGGCCTCTAAACGGGTCTTGAGGGGTTTTTTGCTGAAAGGAGGAACTATATCCGGATTGGCGAATGGGACGC

GCCCTGTAGCGGCGCATTAAGCGCGGCGGGTGTGGTGGTTACGCGCAGCGTGACCGCTACACTTGCCAGCGCCCTAGCGCCCGCTCCTTTCGCTTTCTTCCCTTCCTTTC

TCGCCACGTTCGCCGGCTTTCCCCGTCAAGCTCTAAATCGGGGGCTCCCTTTAGGGTTCCGATTTAGTGCTTTACGGCACCTCGACCCCAAAAAACTTGATTAGGGTGAT

GGTTCACGTAGTGGGCCATCGCCCTGATAGACGGTTTTTCGCCCTTTGACGTTGGAGTCCACGTTCTTTAATAGTGGACTCTTGTTCCAAACTGGAACAACACTCAACCC

TATCTCGGTCTATTCTTTTGATTTATAAGGGATTTTGCCGATTTCGGCCTATTGGTTAAAAAATGAGCTGATTTAACAAAAATTTAACGCGAATTTTAACAAAATATTAAC

GCTTACAATTTAGGTGGCACTTTTCGGGGAAATGTGCGCGGAACCCCTATTTGTTTATTTTTCTAAATACATTCAAATATGTATCCGCTCATGAATTAATTCTTAGAAAAA

CTCATCGAGCATCAAATGAAACTGCAATTTATTCATATCAGGATTATCAATACCATATTTTTGAAAAAGCCGTTTCTGTAATGAAGGAGAAAACTCACCGAGGCAGTTC

CATAGGATGGCAAGATCCTGGTATCGGTCTGCGATTCCGACTCGTCCAACATCAATACAACCTATTAATTTCCCCTCGTCAAAAATAAGGTTATCAAGTGAGAAATCAC

CATGAGTGACGACTGAATCCGGTGAGAATGGCAAAAGTTTATGCATTTCTTTCCAGACTTGTTCAACAGGCCAGCCATTACGCTCGTCATCAAAATCACTCGCATCAAC

CAAACCGTTATTCATTCGTGATTGCGCCTGAGCGAGACGAAATACGCGATCGCTGTTAAAAGGACAATTACAAACAGGAATCGAATGCAACCGGCGCAGGAACACTG

CCAGCGCATCAACAATATTTTCACCTGAATCAGGATATTCTTCTAATACCTGGAATGCTGTTTTCCCGGGGATCGCAGTGGTGAGTAACCATGCATCATCAGGAGTACG

GATAAAATGCTTGATGGTCGGAAGAGGCATAAATTCCGTCAGCCAGTTTAGTCTGACCATCTCATCTGTAACATCATTGGCAACGCTACCTTTGCCATGTTTCAGAAAC

AACTCTGGCGCATCGGGCTTCCCATACAATCGATAGATTGTCGCACCTGATTGCCCGACATTATCGCGAGCCCATTTATACCCATATAAATCAGCATCCATGTTGGAATT

TAATCGCGGCCTAGAGCAAGACGTTTCCCGTTGAATATGGCTCATAACACCCCTTGTATTACTGTTTATGTAAGCAGACAGTTTTATTGTTCATGACCAAAATCCCTTAA

CGTGAGTTTTCGTTCCACTGAGCGTCAGACCCCGTAGAAAAGATCAAAGGATCTTCTTGAGATCCTTTTTTTCTGCGCGTAATCTGCTGCTTGCAAACAAAAAAACCACC

GCTACCAGCGGTGGTTTGTTTGCCGGATCAAGAGCTACCAACTCTTTTTCCGAAGGTAACTGGCTTCAGCAGAGCGCAGATACCAAATACTGTCCTTCTAGTGTAGCCG

TAGTTAGGCCACCACTTCAAGAACTCTGTAGCACCGCCTACATACCTCGCTCTGCTAATCCTGTTACCAGTGGCTGCTGCCAGTGGCGATAAGTCGTGTCTTACCGGGTT

GGACTCAAGACGATAGTTACCGGATAAGGCGCAGCGGTCGGGCTGAACGGGGGGTTCGTGCACACAGCCCAGCTTGGAGCGAACGACCTACACCGAACTGAGATACC

TACAGCGTGAGCTATGAGAAAGCGCCACGCTTCCCGAAGGGAGAAAGGCGGACAGGTATCCGGTAAGCGGCAGGGTCGGAACAGGAGAGCGCACGAGGGAGCTTCC

AGGGGGAAACGCCTGGTATCTTTATAGTCCTGTCGGGTTTCGCCACCTCTGACTTGAGCGTCGATTTTTGTGATGCTCGTCAGGGGGGCGGAGCCTATGGAAAAACGCC

AGCAACGCGGCCTTTTTACGGTTCCTGGCCTTTTGCTGGCCTTTTGCTCACATGTTCTTTCCTGCGTTATCCCCTGATTCTGTGGATAACCGTATTACCGCCTTTGAGTGAG

CTGATACCGCTCGCCGCAGCCGAACGACCGAGCGCAGCGAGTCAGTGAGCGAGGAAGCGGAAGAGCGCCTGATGCGGTATTTTCTCCTTACGCATCTGTGCGGTATTT

CACACCGCAATGGTGCACTCTCAGTACAATCTGCTCTGATGCCGCATAGTTAAGCCAGTATACACTCCGCTATCGCTACGTGACTGGGTCATGGCTGCGCCCCGACACC

CGCCAACACCCGCTGACGCGCCCTGACGGGCTTGTCTGCTCCCGGCATCCGCTTACAGACAAGCTGTGACCGTCTCCGGGAGCTGCATGTGTCAGAGGTTTTCACCGTC

ATCACCGAAACGCGCGAGGCAGCTGCGGTAAAGCTCATCAGCGTGGTCGTGAAGCGATTCACAGATGTCTGCCTGTTCATCCGCGTCCAGCTCGTTGAGTTTCTCCAGA

AGCGTTAATGTCTGGCTTCTGATAAAGCGGGCCATGTTAAGGGCGGTTTTTTCCTGTTTGGTCACTGATGCCTCCGTGTAAGGGGGATTTCTGTTCATGGGGGTAATGAT

ACCGATGAAACGAGAGAGGATGCTCACGATACGGGTTACTGATGATGAACATGCCCGGTTACTGGAACGTTGTGAGGGTAAACAACTGGCGGTATGGATGCGGCGGG

ACCAGAGAAAAATCACTCAGGGTCAATGCCAGCGCTTCGTTAATACAGATGTAGGTGTTCCACAGGGTAGCCAGCAGCATCCTGCGATGCAGATCCGGAACATAATG

GTGCAGGGCGCTGACTTCCGCGTTTCCAGACTTTACGAAACACGGAAACCGAAGACCATTCATGTTGTTGCTCAGGTCGCAGACGTTTTGCAGCAGCAGTCGCTTCACG

TTCGCTCGCGTATCGGTGATTCATTCTGCTAACCAGTAAGGCAACCCCGCCAGCCTAGCCGGGTCCTCAACGACAGGAGCACGATCATGCGCACCCGTGGGGCCGCCA

TGCCGGCGATAATGGCCTGCTTCTCGCCGAAACGTTTGGTGGCGGGACCAGTGACGAAGGCTTGAGCGAGGGCGTGCAAGATTCCGAATACCGCAAGCGACAGGCCG

ATCATCGTCGCGCTCCAGCGAAAGCGGTCCTCGCCGAAAATGACCCAGAGCGCTGCCGGCACCTGTCCTACGAGTTGCATGATAAAGAAGACAGTCATAAGTGCGGC

GACGATAGTCATGCCCCGCGCCCACCGGAAGGAGCTGACTGGGTTGAAGGCTCTCAAGGGCATCGGTCGAGATCCCGGTGCCTAATGAGTGAGCTAACTTACATTAAT

TGCGTTGCGCTCACTGCCCGCTTTCCAGTCGGGAAACCTGTCGTGCCAGCTGCATTAATGAATCGGCCAACGCGCGGGGAGAGGCGGTTTGCGTATTGGGCGCCAGGGT

GGTTTTTCTTTTCACCAGTGAGACGGGCAACAGCTGATTGCCCTTCACCGCCTGGCCCTGAGAGAGTTGCAGCAAGCGGTCCACGCTGGTTTGCCCCAGCAGGCGAAAA

TCCTGTTTGATGGTGGTTAACGGCGGGATATAACATGAGCTGTCTTCGGTATCGTCGTATCCCACTACCGAGATGTCCGCACCAACGCGCAGCCCGGACTCGGTAATGG

CGCGCATTGCGCCCAGCGCCATCTGATCGTTGGCAACCAGCATCGCAGTGGGAACGATGCCCTCATTCAGCATTTGCATGGTTTGTTGAAAACCGGACATGGCACTCCA

GTCGCCTTCCCGTTCCGCTATCGGCTGAATTTGATTGCGAGTGAGATATTTATGCCAGCCAGCCAGACGCAGACGCGCCGAGACAGAACTTAATGGGCCCGCTAACAG

CGCGATTTGCTGGTGACCCAATGCGACCAGATGCTCCACGCCCAGTCGCGTACCGTCTTCATGGGAGAAAATAATACTGTTGATGGGTGTCTGGTCAGAGACATCAAG

AAATAACGCCGGAACATTAGTGCAGGCAGCTTCCACAGCAATGGCATCCTGGTCATCCAGCGGATAGTTAATGATCAGCCCACTGACGCGTTGCGCGAGAAGATTGTG

CACCGCCGCTTTACAGGCTTCGACGCCGCTTCGTTCTACCATCGACACCACCACGCTGGCACCCAGTTGATCGGCGCGAGATTTAATCGCCGCGACAATTTGCGACGGC

GCGTGCAGGGCCAGACTGGAGGTGGCAACGCCAATCAGCAACGACTGTTTGCCCGCCAGTTGTTGTGCCACGCGGTTGGGAATGTAATTCAGCTCCGCCATCGCCGCTT

CCACTTTTTCCCGCGTTTTCGCAGAAACGTGGCTGGCCTGGTTCACCACGCGGGAAACGGTCTGATAAGAGACACCGGCATACTCTGCGACATCGTATAACGTTACTGG

TTTCACATTCACCACCCTGAATTGACTCTCTTCCGGGCGCTATCATGCCATACCGCGAAAGGTTTTGCGCCATTCGATGGTGTCCGGGATCTCGACGCTCTCCCTTATGCG

ACTCCTGCATTAGGAAGCAGCCCAGTAGTAGGTTGAGGCCGTTGAGCACCGCCGCCGCAAGGAATGGTGCATGCAAGGAGATGGCGCCCAACAGTCCCCCGGCCACG

GGGCCTGCCACCATACCCACGCCGAAACAAGCGCTCATGAGCCCGAAGTGGCGAGCCCGATCTTCCCCATCGGTGATGTCGGCGATATAGGCGCCAGCAACCGCACC

TGTGGCG
